# Modified Cryotherapy During Final Irrigation Reduces Postoperative Pain and Anxiety in Pediatric Primary Molar Root Canal Therapy

**DOI:** 10.1002/cre2.70369

**Published:** 2026-04-28

**Authors:** Ting Zhang, Hui‐hui Wang, Liu‐hui Liu, Qing Wu, Wen‐wei Hong, Gui‐lan Fei, Hai‐feng Tang

**Affiliations:** ^1^ Department of Stomatology, Shanghai Fifth People's Hospital Fudan University Shanghai China; ^2^ Department of Stomatology Ma Qiao Community Health Service Centre Shanghai China

**Keywords:** anxiety control, cryotherapy, postoperative pain, primary molars

## Abstract

**Objectives:**

Postoperative pain and dental anxiety are significant barriers to effective pediatric endodontic treatment. Improving patient comfort and cooperation remains a critical clinical objective in school‐age children undergoing primary molar root canal therapy (RCTP). Conventional pharmacologic strategies have limitations in acceptability, safety, and efficacy, prompting the exploration of non‐invasive alternatives. The aim of this study is to evaluate the effect of modified cryotherapy using 0°C saline as the final irrigation protocol on postoperative pain levels and anxiety control in school‐age children undergoing RCTP.

**Material and Methods:**

This single‐center, randomized, controlled clinical trial enrolled 104 school‐age patients diagnosed with pulp or periapical disease in deciduous molars. Patients were randomly allocated to either a modified cryotherapy group or a control group. Pain levels were assessed using the Wong‐Baker FACES Pain Rating Scale (WB Scale) at baseline, 6, 24, 48 h, and 7 days postoperatively. Anxiety and cooperation were evaluated at baseline and 7‐day follow‐up using the Venham Scale. Statistical analyses included Chi‐square and Mann–Whitney *U* tests, with significance at *p* < 0.05.

**Results:**

Baseline characteristics were comparable between groups. The cryotherapy group showed significantly lower pain scores at 6 h,24 h, and 48 h (*p* <  0.001) post‐treatment compared to controls. At 7 days, pain levels converged with no significant difference. The cryotherapy group also demonstrated significantly improved anxiety scores (*p* = 0.005) and clinical cooperation (*p*  = 0.012) at follow‐up compared to the control group.

**Conclusion:**

Modified cryotherapy applied during final root canal irrigation significantly reduces short‐term postoperative pain and enhances clinical cooperation in school‐age children.

## 1 Introduction

Root canal therapy in primary molars (RCTP) has been employed since 1932 to manage pulp and periapical diseases caused by carious and non‐carious factors (Burns et al. 2022). Over time, accumulating clinical evidence has demonstrated that RCTP yields better long‐term outcomes in deciduous molars than alternative treatments (Praveen et al. 2017; Yildirim et al. 2017). By preserving affected primary teeth until the mixed dentition phase, RCTP facilitates the maintenance of arch integrity, contributes to proper occlusal development, and supports sustained oral hygiene. As a result, RCTP is now considered the standard therapeutic approach for managing pulp and periapical pathology in primary molars (Burns et al. 2022; Yildirim et al. 2017).

Children in the mixed dentition stage (typically 7–12 years) exhibit improved cognitive abilities and greater awareness of clinical environments. However, this developmental stage is also associated with heightened sensitivity to pain, increased anxiety, and reduced tolerance to dental procedures (Carneiro et al. 2022; Cademartori et al. 2017; Azher et al. 2020). These psychological factors frequently result in diminished cooperation, negatively impacting treatment outcomes and clinical efficiency.

Vera et al. (2015, 2018) introduced cryotherapy into adult root canal therapy by incorporating 2.5°C sodium chloride irrigation using the EndoVac negative pressure system (Kerr Corporation, Brea, CA, USA). This protocol, involving apical irrigation at a controlled flow rate of 4 mL/min for 5 min, has since been adopted in clinical studies and validated by systematic reviews as an effective method for reducing postoperative pain and analgesic use in adult endodontic patients (Al‐Nahlawi et al. 2016; Alharthi et al. 2019; Sadaf et al. 2020). Despite its proven efficacy in adults, the applicability and effectiveness of cryotherapy in pediatric endodontics remain unclear. Furthermore, previous cryotherapy protocols require specialized equipment and temperature control systems, which limit their feasibility in routine pediatric practice (Kaplan et al. 2022; Hamza et al. 2024; Solomon et al. 2024).

The present study investigates a simplified cryotherapy approach for pediatric patients by modifying existing irrigation protocols to address this gap. This includes preparing cold saline (0°C) via an ice‐water bath and manually delivering it with a dual‐open‐ended irrigation needle positioned 1 mm short of the working length. Through a randomized controlled trial involving non‐invasive pain and anxiety assessment tools, we aim to determine whether this technique can reduce short‐term postoperative discomfort and improve clinical cooperation in school‐age children undergoing RCTP. The study seeks to provide practical evidence to support the integration of cryotherapy into behaviorally optimized pediatric dental protocols.

## 2 Methods

### 2.1 Study Design and Ethical Approval

This single‐center, randomized controlled trial was conducted at the Department of Dentistry, Shanghai Fifth People's Hospital, from January 2023 to February 2025. The study was conducted by the Declaration of Helsinki and approved by the Medical Ethics Committee of Shanghai Fifth People's Hospital, affiliated with Fudan University (Approval No.: 2023 Ethics Review 163). The trial was registered on the National Health Security Information Platform (Registration No.: MR‐31‐24‐024259; URL: www.medicalresearch.org.cn). Informed consent was obtained from all participating patients and their legal guardians.

### 2.2 Participants

**Figure 1:**
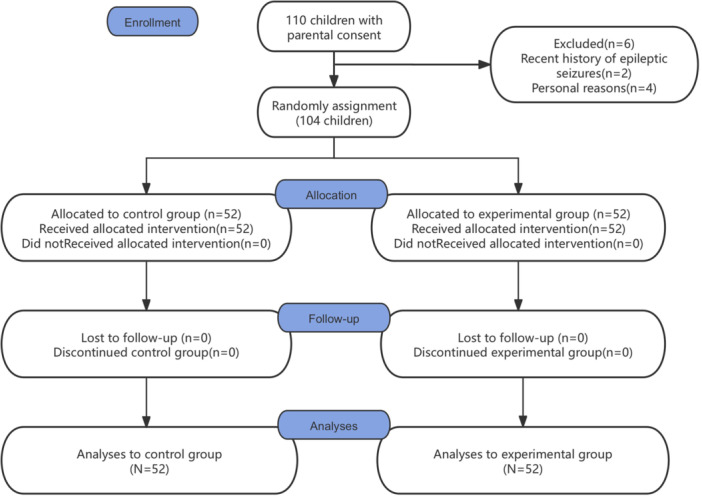
Flow of participants: 110 patients screened for this study, 6 exclusions occurred: 2 cases were excluded based on recent seizure episodes, and 4 based on guardians' personal reasons. The remaining 104 patients were randomly allocated into two groups, with all participants completing the study interventions and follow‐up assessments through the study endpoint.

A total of 110 school‐age children (7–12 years) diagnosed with pulp or periapical diseases in primary molars were initially recruited. Two were excluded due to recent epileptic seizures, and four withdrew for personal reasons. The remaining 104 participants completed the study and were included in the final analysis, with 52 children allocated to each group using a block randomization method (Figure 1). Block randomization was adopted during the trial design phase based on the anticipated similarity in the baseline characteristics of the intended patient population. This methodological choice is supported by the data presented in Table 1. In detail, block sizes of 6 and 8 were used alternately. There were 7 complete blocks, and the remaining 6 cases were treated as an additional block, with 3 randomly assigned to the experimental group and 3 to the control group.

**Table 1:** Baseline characteristics and preoperative assessment.

Title	Designation	Group (%)		Total	*χ* ^2^/*t*	*p*
		Experimental group	Control group			
Gender	Female	26 (50.000)	20 (38.462)	46 (44.231)	1.403	0.236
	Male	26 (50.000)	32 (61.538)	58 (55.769)		
Affected tooth site	Upper right	13 (25.000)	10 (19.231)	23 (22.115)	1.952	0.582
	Lower right	18 (34.615)	14 (26.923)	32 (30.769)		
	Upper left	9 (17.308)	13 (25.000)	22 (21.154)		
	Lower left	12 (23.077)	15 (28.846)	27 (25.962)		
Pathogenic factors	Non‐carious	3 (5.769)	2 (3.846)	5 (4.808)	0.210	0.647
	Carious	49 (94.231)	50 (96.154)	99 (95.192)		
Extent of involvement	Apex	21 (41.176)	23 (44.231)	44 (42.718)	0.098	0.754
	Dental pulp	30 (58.824)	29 (55.769)	59 (57.282)		
Age		8.308 ± 0.829	8.462 ± 0.979		−0.864	0.389
Venham initial Consultation		3.000 (2.000,4.000)	3.000 (2.000,3.750)		−0.273	0.785
WB preoperative		7.000 (4.500,8.500)	7.000 (5.125,7.500)		−0.591	0.554

#### 2.2.1 Inclusion Criteria

1. Primary molars with irreversible pulpitis or pulp necrosis due to caries or trauma.
2. Chronic periapical lesions without acute suppuration and with radiographic evidence of root resorption are limited to the apical third.
3. Teeth previously drained for acute suppurative periodontitis that had transitioned to the chronic phase, with similar radiographic findings.

#### 2.2.2 Exclusion Criteria

1. Need for root canal retreatment or presence of perforations.
2. Internal or external root resorption exceeds the apical third.
3. Extensive apical or furcation bone loss with the involvement of permanent tooth germs.
4. Acute apical abscess or space infections requiring drainage.
5. Systemic illness, long‐term medication use, or history of epilepsy.
6. Refusal to participate or sign informed consent.

### 2.3 Intervention and Blinding

Due to the irrigant perceptible temperature, blinding operators was not feasible. However, patients were blinded to group allocation due to rubber dam isolation. Randomization was performed using a stratified block method. If a patient had multiple eligible molars, one tooth was treated first, and subsequent treatments were re‐randomized. During the trial design phase, we considered the possibility that the same patient might have multiple eligible primary molars and established a corresponding handling strategy. However, this situation did not materialize in the actual study. After randomization, clinicians performing the procedures were experienced pediatric and endodontic specialists. Outcome assessors and data analysts were blinded to group assignment.

### 2.4 Clinical Procedure

Preoperative behavior management was conducted using the Tell‐Show‐Do technique. Baseline anxiety and cooperation were assessed using the Venham Scale. Following routine oral disinfection, local anesthesia was administered (1.6 mL of 4% articaine with 1:200,000 epinephrine) (ACTEON Group, Lyon, France). Affected teeth were isolated with a rubber dam. Access was achieved using standard high‐ and low‐speed handpieces, and working length was determined using preoperative radiographs in combination with 25# K‐file probing (Dentsply Tulsa Dental Specialties, Tulsa, OK, USA). Root canal instrumentation was performed with SANI S3 pediatric rotary files (sizes 20, 25, 30) at 500 rpm and 2.5 N·cm torque (SANI Medical Equipment Co. Chengdu, China). Irrigation involved alternating 17% EDTA (META BIOMED, Cheongju, Korea), 1.5% sodium hypochlorite (Longly Biotechnology, Wuhan, China), and 0.9% saline (Kelun Industry Group, Chengdu, China), each delivered at 4 mL/min for 5 min.

#### 2.4.1 Experimental and Control Groups

• **Experimental group:** Received final irrigation with 20 mL of 0°C 0.9% saline using a dual‐open‐ended irrigation needle positioned 1 mm short of the working length (4 mL/min over 5 min). The irrigation needle was made of a polytetrafluoroethylene material, which is soft and flexible, with a total working length of 21 mm, a distal diameter of 0.27 mm, and a taper of 4%(Baiqi Medical, Dongguan, China).
• **Control group:** Received 20 mL of room‐temperature 0.9% saline using the same technique.

Following irrigation, canals were dried with sterile paper points, filled with calcium hydroxide paste, and sealed with glass ionomer cement. Patients were recalled after 7 days for obturation and restoration.

A total of 1.5% sodium hypochlorite was selected as the root canal irrigant, even though a rubber dam was used throughout the entire treatment. Consequently, to control infection as effectively as possible, calcium hydroxide paste was applied for intracanal medication. In fact, for the coronal seal during the initial visit, glass ionomer (FX‑II, Shofu, Japan)—a material suitable for permanent restoration of primary molars—was used as the filling material. To minimize potential pain/trauma for the patient and to lower treatment costs, at the 7‑day follow‑up, the canals were obturated under rubber dam isolation using a calcium hydroxide‑based root canal filling material (Vitapex, Morita, Japan). For the final coronal restoration, composite resin (BEAUTIFIL II, Shofu, Japan) was chosen as the permanent restoration instead of alternatives such as stainless steel preformed crowns.

### 2.5 Pain and Anxiety Assessment

Postoperative pain was assessed using the Wong‐Baker FACES Pain Rating Scale (WB Scale) at baseline, 6, 24, 48 h, and 7 days postoperatively (Keck et al. 1996). The WB scale ranges from 0 (no pain) to 10 (worst pain), with six facial expressions corresponding to intensity scores:

0–2 = Grade I (mild)

2–4 = Grade II (mild)

4–6 = Grade III (moderate)

6–8 = Grade IV (severe)

8–10 = Grade V (intolerable)

Clinical anxiety and cooperation were assessed using the Venham Scale preoperatively and at the 7‐day follow‐up (Venham et al. 1980). Assessments were performed by research staff at two time points: prior to treatment initiation and at the 7‐day follow‐up visit, with no parental training required. To mitigate assessment bias, the two evaluations were conducted independently by different researchers. The scale categorizes patient behavior into grades 0 (comfortable) to 5 (extreme fear), with grades 0–2 considered cooperative and grades 3–5 non‐cooperative. Clinical cooperation rate = cooperative cases/total cases.

### 2.6 Sample Size Calculation

Sample size was calculated using PASS software based on a 1:1 superiority design. Based on existing literature results, we separately estimated sample sizes required for primary evaluation metrics between experimental and control groups (Vera et al. 2018; Sadaf et al. 2020). Using WB Scale data at 6 h post‐treatment (mean ± SD: 1.59  ±  1.9 in the test group vs. 3.53  ±  1.9 in controls) and assuming an expected difference (Δ) of 1, with a significance level of *α* = 0.05 and power of 80%, the required sample size was approximately 45 participants per group. Considering that the study population consisted of patients in the mixed dentition stage, a dropout rate of 20% was anticipated, a total of 110 participants were enrolled.

### 2.7 Statistical Analysis

Data were analyzed using SPSS 29.0 software (SPSS Inc., Chicago, IL, USA). The Shapiro–Wilk test assessed normality. Parametric data were expressed as mean ± SD and compared using *t*‐tests or Chi‐square tests. Ordinal outcomes (WB Scale and Venham Scale scores) were analyzed using cross‐tabulation Chi‐square tests. Changes in cooperation levels were assessed using the Mann–Whitney *U* test. A *p*‐value < 0.05 was considered statistically significant.

## 3 Results

### 3.1 Baseline Characteristics and Preoperative Assessment

Table 1 shows no statistically significant differences between the experimental and control groups concerning baseline characteristics, including gender distribution, tooth location, etiological factors, lesion extent, and patient age (*p* > 0.05). Additionally, preoperative anxiety levels assessed using the Venham Scale and pain levels evaluated using the Wong‐Baker FACES Pain Rating Scale (WB Scale) showed no significant differences between the two groups (*p* > 0.05). These findings confirm that both groups were comparable at baseline concerning demographic and clinical parameters.

### 3.2 Preoperative and Postoperative Pain Comparison

Pain levels were evaluated using the WB Scale at five time points: baseline, 6, 24, 48 h, and 7 days postoperatively. Table 2 shows that no statistically significant difference between the experimental and control groups was observed in preoperative pain levels (*p* > 0.05). At 6, 24, and 48 h post‐treatment, the experimental group demonstrated significantly lower pain scores than the control group (*p* < 0.05). At the 7‐day follow‐up, pain levels had decreased in both groups, with no significant intergroup difference (*p* > 0.05). Overall, postoperative pain scores improved over time in both groups, but pain reduction was more pronounced in the experimental group during the early postoperative period.

**Table 2:** Preoperative and postoperative pain comparison.

WB scale	Grade	Experimental group	Control group	Total	*χ* ^2^	*p*
Preoperative	Grade II	7 (13.462)	4 (7.692)	11 (10.577)	3.492	0.322
	Grade III	10 (19.231)	13 (25.000)	23 (22.115)		
	Grade IV	20 (38.462)	26 (50.000)	46 (44.231)		
	Grade V	15 (28.846)	9 (17.308)	24 (23.077)		
6 h postoperatively	Grade I	20 (38.462)	0 (0.000)	20 (19.231)	59.077	0.000**
	Grade II	4 (7.692)	20 (38.462)	24 (23.077)		
	Grade III	28 (53.846)	11 (21.154)	39 (37.500)		
	Grade IV	0 (0.000)	20 (38.462)	20 (19.231)		
	Grade V	0 (0.000)	1 (1.923)	1 (0.962)		
24 h postoperatively	Grade I	41 (78.846)	3 (5.769)	44 (42.308)	67.434	0.000**
	Grade II	11 (21.154)	15 (28.846)	26 (25.000)		
	Grade III	0 (0.000)	28 (53.846)	28 (26.923)		
	Grade IV	0 (0.000)	6 (11.538)	6 (5.769)		
48 h postoperatively	Grade I	50 (96.154)	32 (61.538)	82 (78.846)	18.793	0.000**
	Grade II	2 (3.846)	17 (32.692)	19 (18.269)		
	Grade III	0 (0.000)	3 (5.769)	3 (2.885)		
7 days postoperatively	Grade I	38 (73.077)	25 (48.077)	63 (60.577)	8.483	0.075
	Grade II	7 (13.462)	13 (25.000)	20 (19.231)		
	Grade III	3 (5.769)	9 (17.308)	12 (11.538)		
	Grade IV	4 (7.692)	4 (7.692)	8 (7.692)		
	Grade V	0 (0.000)	1 (1.923)	1 (0.962)		

** *p* < 0.01.

### 3.3 Anxiety and Clinical Cooperation Comparison

Anxiety levels and clinical cooperation were assessed using the Venham Scale during the initial consultation and the 7‐day follow‐up (Table 3). At baseline, no statistically significant differences existed between groups in anxiety levels or cooperation scores (*p* > 0.05). At the 7‐day recall visit, the experimental group demonstrated significantly lower anxiety levels (*p* < 0.05) and improved cooperation compared to the control group (*p* < 0.05). These findings suggest that cryotherapy was associated with improved behavioral outcomes following treatment.

**Table 3:** Anxiety and clinical cooperation comparison.

Venham scale/cooperation		Experimental group (*n* = 52)	Control group (*n* = 52)	*χ* ^2^/*z*	*p*
Venham initial consultation		3.000 (2.000,4.000)	3.000 (2.000,3.750)	−0.273	0.785
Venham follow‐up visit		1.000 (0.000,2.000)	2.000 (1.000,3.000)	−2.784	0.005**
Initial consultation cooperation	Cooperation	19 (36.538)	19 (36.538)	0	1
	Non‐cooperation	33 (63.462)	33 (63.462)		
Follow‐up visit cooperation	Cooperation	45 (86.538)	34 (65.385)	6.372	0.012*
	Non‐cooperation	7 (13.462)	18 (34.615)		

* *p* < 0.05.

** *p* < 0.01.

## 4 Discussion

This randomized controlled trial demonstrated that modified cryotherapy using 0°C saline as the final irrigant during RCTP significantly reduced postoperative pain at 6, 24, and 48 h compared to conventional irrigation. Additionally, anxiety levels and clinical cooperation, as measured during follow‐up visits, were significantly improved in the cryotherapy group. Baseline characteristics—including age, gender, lesion extent, and preoperative anxiety—were statistically comparable across groups, confirming that observed improvements were attributable to the cryotherapy intervention.

Upon analyzing the results, we found that there was no statistically significant difference in pain levels across grades between the experimental and control groups preoperatively (*p* > 0.05). Since no treatment or related interventions had been administered preoperatively, the pain severity was Grade II or above in all cases. Statistically significant differences in pain levels were observed between the experimental and control groups at 6, 24, and 48 h postoperatively (*p* < 0.05). In detail, at 6 h postoperatively, no patients in the experimental group reported Grade IV or V pain, whereas in the control group, 20 cases (38.462%) reported Grade IV and 1 case (1.923%) reported Grade V pain. At 24 and 48 h postoperatively, pain grades decreased in both groups. In the experimental group, pain was limited to Grades I and II. In the control group at 24 h, 28 cases (53.846%) reported Grade III pain and 6 cases (11.538%) reported Grade IV pain; by 48 h, only 3 cases (5.769%) reported Grade III pain. By postoperative day 7, the pain grades were concentrated in Grade I (Experimental group: 38; Control group: 25; collectively accounting for 60.577% of cases) and Grade II (Experimental group: 7; Control group: 13; collectively accounting for 19.231% of cases). Therefore, at the 7‐day follow‐up, pain levels had decreased in both groups, with no significant intergroup difference (*p* > 0.05) and pain reduction was more pronounced in the experimental group during the early postoperative period.

During data collation, it was observed that patients who received high‐grade Venham scores (Grade 5: extreme fear) during the initial visit also obtained high scores on the WB scale, categorized as Grade IV or V. At this stage, patients were unable to communicate normally or describe their symptoms due to extreme fear. We propose that the patient's pain and anxiety stem not only from the affected tooth itself but also from a deeper‐seated fear of the hospital environment and the symbolic “white coat,” potentially influenced by cultural products such as literature, films, and comics (Dunkeld et al. 2020; Wanniarachchi et al. 2025). Consequently, while pain levels significantly decreased in WB scale assessments at 6, 24, and 48 h postoperatively, a notable resurgence of negative emotions (Grade IV) was observed during the 7‐day follow‐up visit, likely triggered by the return to the clinical environment.

The findings align with previous adult studies demonstrating the analgesic effect of apical cryotherapy during endodontic procedures (Vera et al. 2018; Sadaf et al. 2020). However, this study is among the first to extend these observations to a pediatric population, where fear and behavioral resistance are more prevalent. Prior research has primarily focused on pharmacologic methods, including NSAIDs and sedative protocols, which may not be ideal for pediatric use due to side effects or low parental acceptance (Mathur et al. 2024; Aggarwal et al. 2024; Nosrat et al. 2021). By comparison, our simplified cryotherapy method offers a non‐pharmacological alternative with favorable safety and acceptability profiles in children. These findings also align with future research directions in oral health science (Klingberg and Broberg 2010; Zhang et al. 2020).

Three irrigants were used as chemical adjuncts during root canal preparation in this study: 17% EDTA, 1.5% sodium hypochlorite (NaClO), and 0.9% saline (Duncan et al. 2023; Zou et al. 2024). The 17% EDTA solution, which removes the smear layer and exposes dentinal tubules by chelating inorganic components, was used alternately with 1.5% NaClO after each session of mechanical instrumentation but was not included in the final cycle of the end‐irrigation sequence. Following the last application of 17% EDTA, the canals were initially rinsed with 5 mL of 0.9% saline to displace residual EDTA, followed by a 10 mL rinse with 1.5% NaClO. The procedure then entered the final end‐irrigation cycle, which was conducted using 0.9% saline (applied to both experimental and control groups).

This study modified the traditional cryotherapy protocol by replacing complex temperature‐controlled delivery systems with a readily accessible ice‐water method. A dual‐open‐ended irrigation needle allowed controlled delivery 1 mm short of the working length, enhancing irrigation efficacy while maintaining simplicity. This protocol addresses prior barriers to clinical adoption, such as cost, device complexity, and time requirements. The improved behavioral outcomes observed may be attributed to cryotherapy's physiologic effect on nerve conduction and local inflammation, which may reduce nociceptor activation and pain‐associated anxiety.

The Venham Scale was introduced as early as the 1980s to describe and assess patient anxiety and cooperation based on observed behaviors and responses (Venham et al. 1980). Since its development by Wong and Baker (1988), the Wong‐Baker FACES Scale (WB Scale) has been widely used in pediatric dentistry and clinical research, despite some questioning and challenges (Wong and Baker 2026). For instance, it has been employed in pain assessment following the application of new technologies or devices such as trace image and coloring books for kids and 3D‐printed hip fixators (Suleman et al. 2023; Zeng et al. 2024). These tools are widely used in pediatric dentistry and contribute to the reliability of our findings.

Based on the definition provided in the reference text, the age range for inclusion in this study was set at 7–12 years, as it focuses on school‐aged children (Dean et al. 2015). However, a retrospective review of the collected data revealed that all enrolled patients were under 12 years of age. Throughout the study period, patients aged 12 or older were generally not included because their deciduous molars had either exfoliated naturally or were in the process of replacement, with successor permanent teeth already erupting; any remaining teeth in this age group were considered over‐retained and thus excluded. Among the enrolled participants, only one 11‐year‐old male was included. In the 10‐year‐old cohort, males also predominated, with 11 participants compared to only 1 female participant. Additionally, the baseline characteristics of enrolled patients included age, gender, affected tooth site, pathogenic factors, and extent of lesion. While other studies may incorporate factors such as place of birth, guardian's age range and educational level, family size, and annual household income for subgroup analysis, these variables were not included in the present research (Chiu et al. 2025; Eric et al. 2025). This decision was made considering the limited sample size of the study, as excessive subgroup analyses could reduce the precision of pooled estimates and potentially lead to false‐positive findings inconsistent with the overall results. Future studies with larger cohorts could incorporate these variables into subgroup analyses.

This study focused exclusively on school‐age children capable of providing feedback and demonstrating observable behaviors. Its findings may not extend to preschool‐aged children or those with developmental limitations. Further studies are warranted to evaluate the feasibility and effectiveness of modified cryotherapy in younger cohorts, where behavioral management poses greater challenges.

In conclusion, modified cryotherapy using cold saline irrigation significantly reduced postoperative pain and improved clinical cooperation and anxiety outcomes in school‐age children undergoing RCTP. The simplified delivery method is cost‐effective, easy to implement, and non‐invasive. These findings support the inclusion of cryotherapy in behaviorally sensitive pediatric endodontic protocols and provide a foundation for developing standardized pain management frameworks in pediatric dental care.

## Author Contributions

Substantial contributions to the conception, design, execution, analysis, and/or interpretation of the study, as well as to drafting or critically revising the manuscript, were made by each author. All authors undertook significant responsibility for the intellectual content and quality of the work. A collective review and endorsement of the final manuscript was undertaken by all authors, who affirm their agreement with its content and commit to its submission for publication as presented.

## Ethics Statement

The study was conducted by the Declaration of Helsinki and approved by the Medical Ethics Committee of Shanghai Fifth People's Hospital, affiliated with Fudan University (Approval No.: 2023 Ethics Review 163).

## Consent

Informed consent was obtained from all participating patients and their legal guardians.

## Conflicts of Interest

The authors declare no conflicts of interest.

## Data Availability

The data that support the findings of this study are available on request from the corresponding author. The data are not publicly available due to privacy or ethical restrictions.
